# The Lived Care Experiences of Neurorehabilitation Inpatients: A Phenomenological Qualitative Exploratory Study

**DOI:** 10.3390/clinpract16100177

**Published:** 2026-09-24

**Authors:** Carlo Alberto Camuccio, Valeria Basso, Sara Guglielmi, Laura Danesin, Francesca Burgio, Enrico Dal Pozzo, Luisa Cacciante

**Affiliations:** 1 Immunology and Molecular Oncology Diagnostics Unit, Veneto Institute of Oncology, Via Gattamelata 64, 35128 Padova, Italy; alberto.camuccio@unipd.it; 2 General Surgery, University Hospital of Padua, Via Giustiniani 2, 35128 Padova, Italy; valeria.basso.2@studenti.unipd.it; 3 Intensive Care, AULSS 1 Dolomiti, Via Bagnols Sur Cèzè, 1, 32032 Feltre, Italy; sara.a.guglielmi@gmail.com; 4 Neuropsychology Laboratory, IRCCS San Camillo Hospital, Via Alberoni 70, 30126 Venice, Italy; laura.danesin@hsancamillo.it (L.D.); francesca.burgio@hsancamillo.it (F.B.); 5 IRCCS San Camillo Hospital, Via Alberoni 70, 30126 Venice, Italy; enrico.dalpozzo@hsancamillo.it; 6 Laboratory of Healthcare Innovation Technology, IRCCS San Camillo Hospital, Via Alberoni 70, 30126 Venice, Italy

**Keywords:** neurorehabilitation, therapeutic relationship, inpatient experience, interdisciplinary care, phenomenological approach, emotional support

## Abstract

**Background and Aim:** Hospitalization is increasingly seen as a key factor in rehabilitation outcomes, shaped by patient satisfaction, motivation, and relationships with staff. The lived experience of neurorehabilitation across neurological conditions, such as stroke, multiple sclerosis, Parkinson’s disease, and post-oncological care, remains largely unknown. This study explores the care and hospitalization experience of individuals admitted to neurorehabilitation units with diagnoses of stroke, multiple sclerosis, Parkinson’s disease, and post-neurosurgical recovery following oncological procedures. **Methods:** Thirty-seven patients with neurological conditions were enrolled and interviewed. A descriptive phenomenological design was employed, using semi-structured interviews. Data were analyzed following Colaizzi’s method, supported by Atlas.ti 25. Phenomenological analysis was used to identify themes emerging from patient narratives, with rigor ensured through independent coding, triangulation, and saturation-based sampling. **Results:** Five themes emerged: (1) the therapeutic bond with healthcare professionals; (2) the slow time of hospitalization, marked by waiting and boredom; (3) care is rehabilitation; (4) illness narratives; and (5) stories of closeness among inpatients and family members. **Conclusions**: The results of this study highlight neurorehabilitation as a relational process. These findings reinforce the importance of considering patients’ experiences as an integral component of neurorehabilitation hospitalization. Future research should further explore how these dimensions may contribute to the development of more person-centered approaches to neurorehabilitation.

## 1. Introduction

The hospitalization experience is increasingly recognized as a pivotal factor in determining health outcomes, particularly in the field of rehabilitation. It is becoming evident that aspects such as patient satisfaction, motivation, well-being, relationships with healthcare staff and caregivers, and the social climate of the ward significantly influence the patient’s perception of their care pathway [1,2,3,4].

In neurorehabilitation, the literature consistently highlights the importance of care environment, as well as activities and interpersonal relationships which are not necessarily limited to therapeutic interactions with staff. This body of research is particularly well developed—mainly through qualitative studies—among patients with acquired brain injuries (ABIs), most notably people with stroke (PwS).

Indeed, the recent systematic review by Lafiatoglu et al. on the perception of well-being among older adults with stroke undergoing neurorehabilitation identified five key themes that influence patients’ sense of well-being: rehabilitation processes, issues related to identity and self-perception, institutional factors, the experience of caring, and participation in creative activities during hospitalization [3].

From a different perspective—not specifically focused on well-being but rather on the hospitalization experience itself—the systematic review by Luker et al., which analyzed 31 studies, found that all participants reported negative rehabilitation experiences [4]. Similarly, Kenah and colleagues found feelings of powerlessness, boredom and frustration, lack of control, lack of understanding, and ineffective communication [5], and Rosewilliam et al. reported negative experiences and various barriers that hinder the implementation of a patient-centered goal-setting model in post-stroke rehabilitation practice [6]. Finally, according to Neils-Strunjas et al., resilience plays a vital role in the recovery and rehabilitation process by enhancing individuals’ emotional adjustment and adaptation following ABI [7].

Despite these insights, the lived experience of neurorehabilitation remains underexplored for many neurological conditions. The literature concerning patients with multiple sclerosis (MS) appears more limited. Nevertheless, important contributions by Ghaidar et al. and Giesler et al. highlight a key theme in the hospitalization experience of patients with MS: the lack of, and consequent need for, involvement in decision-making processes regarding their care and rehabilitation [8,9]. A moderate body of research has addressed the lived experiences of individuals with Parkinson’s disease (PD) [10,11], even though qualitative studies specifically focusing on inpatient rehabilitation experiences remain scarce. Within the neuro-oncological field, although previous qualitative studies have explored the experiences of illness and embodied life following neurosurgical intervention [12], as well as language recovery and communication among neuro-oncological patients [13], qualitative evidence specifically addressing the experience of hospitalization within an inpatient neurorehabilitation setting remains limited. Even if these diagnostic groups differ from a clinical and care perspective, they nevertheless share the experience of neurorehabilitation hospitalization, the relationships with healthcare professionals, and the adaptation to hospital routines. While acknowledging the need for clinical and care sensitivity, exploring these experiences across different diagnostic categories may contribute to identifying common experiential dimensions of neurorehabilitation hospitalization that are not specific to a single condition.

To our knowledge, there are no qualitative studies that have explored the experience of hospitalization among different neurorehabilitation populations within the same study, and the experiential dimensions shared across diagnostic groups remain poorly understood.

The aim of this study is to explore and describe the care and hospitalization experience of individuals admitted to neurorehabilitation units with diagnoses of stroke, MS, PD, and post-neurosurgical recovery following oncological procedures.

## 2. Materials and Methods

### 2.1. Study Design

The current study employed a descriptive phenomenological design, following Colaizzi’s method [14]. Phenomenology is a qualitative method that posits that information and insight do not come from large amounts of data, but emerge from an intense study of experiences [14]. This study design allows for the contextualization of the individual lived experiences of patients undergoing neurorehabilitation. This study adopted a reflexive approach based on reciprocal validation of pre-understandings throughout the interpretative process, supported by collaborative coding and phenomenological analysis [14]. The study was registered in ClinicalTrials.gov (NCT06264037) and adheres to the principles of the Declaration of Helsinki. All personal data were pseudonymized and securely stored. The study received ethical approval from the competent territorial ethics committee.

The study was registered on ClinicalTrials.gov on 16 February 2024, after study initiation. This is an observational qualitative study and participants were not assigned to any intervention. For observational studies, registration on ClinicalTrials.gov is permitted after study initiation; the study was registered subsequently for transparency and public accessibility of the study information.

### 2.2. Data Analysis

Semi-structured interviews on hospitalization experience were conducted with patients hospitalized in a neurorehabilitation hospital. The interview data were analyzed with Atlas.ti© software (versions 25) (atlasti.com). This software was used exclusively as a data management tool to support the organization and management of the qualitative data, including transcripts, codes, field notes and analytic memos.

The analysis followed Colaizzi’s [15] seven-step method, as described below:

1. Familiarization: All collected text was transcribed verbatim into a Word file, then read multiple times by the researchers to familiarize themselves with the material and to achieve an overall understanding. The text was then imported into Atlas.ti 25. During this stage, researchers attempted to explore the phenomenon from the participants’ perspectives.
2. Identifying significant statements: Two researchers independently identified quotations deemed significant in describing the phenomenon.
3. Formulating meanings: The two researchers independently formulated meanings from the significant statements, staying close to participants’ original expressions. Meanings were then compared and discussed to ensure consistency and fidelity to the data.
4. Clustering themes: The final coding structure and associated raw data were processed to restore meaning and enhance validity. Differences in coding between the two researchers were discussed by the research team to reach consensus. The final themes were developed through consensus among the research group, avoiding the imposition of pre-existing theoretical frameworks [16].

Phases 3 and 4 also involved the use of field notes and memos (via Atlas.ti 25).

5. Developing an exhaustive description: A comprehensive narrative of the phenomenon was produced by the two researchers.
6. Producing the fundamental structure: A concise and focused statement was formulated, highlighting the essence of the lived experience.
7. Seeking verification of the fundamental structure: This final step, which involves verification of the fundamental structure of the findings with participants, was not undertaken. Whilst acknowledging the epistemological value of member checking, its practical implementation in this specific clinical context was unfeasible. The inpatient neurorehabilitation setting is characterized by dynamic clinical trajectories, including transfers to other healthcare facilities, planned discharges, and fluctuations in patients’ cognitive and physical status. These factors precluded the reliable re-engagement of participants in the review of the interview material.

The research team unanimously agreed to prioritize transparency within the research process by explicitly recognizing this omission as a study limitation, rather than applying a procedural step that could not have been conducted with adequate methodological rigor. Consequently, particular emphasis was placed on strengthening other strategies to ensure trustworthiness, including reflexive discussions, triangulation of data sources, and consensus meetings throughout the analytical process.

### 2.3. Principles of Methodological Rigor and Data Fidelity

To ensure methodological rigor and data fidelity, (a) interviews were audio-recorded and transcribed verbatim; (b) two researchers independently analyzed the interviews; (c) discrepancies in coding were resolved through discussion; (d) triangulation of data sources (i.e., interviews, field notes, and clinical data) was used to enhance credibility. This process of constant comparison supported the credibility and reliability of the findings. Variables related to demographic and clinical data, as well as those related to the Barthel Index, were treated using descriptive statistics.

The study was reported in accordance with the Consolidated Criteria for Reporting Qualitative Research (COREQ) guidelines [17]. The COREQ checklist can be found in the Supplementary Materials, File S1.

### 2.4. Setting and Participants

This study was conducted at a neurorehabilitation hospital in Northern Italy. In 2024, the hospital reported an average length of stay of 79 days when the interviews were conducted and a mean patient age of 60.4 years. A saturation sampling strategy was adopted for the three main diagnostic groups (stroke, MS, PD). The two post-neurosurgical participants were included as illustrative cases, given the rarity of this population, without any claim of achieving saturation within this subgroup. According to Moser & Korstjens [18], “Data saturation means the collection of qualitative data to the point where a sense of closure is attained because new data yield redundant information”. It is not easy to determine the sample size a priori due to the use of saturation-based sampling, but Hennink and Kaiser suggest that valid saturation can be achieved with relatively small samples, typically ranging from 9 to 17 interviews [19]. Saturation was used as a methodological criterion to define the sample, without any claim of exhaustiveness: the analysis valued both recurring themes and unique experiences, in line with the phenomenological approach, which privileges the depth of meaning over frequency.

We included adult (i.e., >18 age and <85 years) Italian-speaking patients who were admitted to the intensive neurorehabilitation unit of the hospital, were hospitalized for ≥21 days without being close to discharge and were able to provide valid informed consent. We excluded patients with severe comprehension deficits, with cognitive or psychiatric conditions preventing the completion of the interview, and patients admitted to the hospital with a diagnosis other than neurological conditions.

### 2.5. Procedure

Patients who met the inclusion criteria were enrolled and, together with the researcher, agreed on a time to conduct the interview, which was audio-recorded upon participants’ consent. The interview took place in a private and quiet environment so that the patient and the researcher were not interrupted and so that the researcher could take field notes. The interviews were conducted in Italian by trained researchers in qualitative studies including both male and female interviewers. C.A.C. is a nurse and sociologist, E.D.P. is a medical anthropologist, and S.G. is a nurse. No individuals other than the participant and the interviewer were present during the interviews, and there were no prior relationships between interviewers and participants, who knew nothing about the researchers other than their occupation. On average, the interviews lasted 30 min.

To test the semi-structured interview grid, three to four pilot interviews were conducted, which were not included in the sample to be analyzed. Given the limited existing research, except for stroke patients in neurorehabilitation, these pilot interviews helped refine the interview grid. It covered several key areas, presented as open-ended questions (Table 1). The interview questions were intentionally formulated in a broad and open-ended manner to allow participants to describe the aspects of hospitalization they considered most meaningful. Given the exploratory nature of the study and the limited qualitative literature on neurorehabilitation hospitalization experiences, a non-directive interviewing approach was adopted to facilitate the emergence of participants’ own perspectives and the meanings they attributed to their experiences.

In addition to the main questions, researchers used probing questions and follow-up prompts to encourage more detailed responses from participants.

Every eight interviews, the researchers conducted a preliminary analysis to assess emerging contents and evaluate data saturation, with the support of Atlas.ti (versions 25).

## 3. Results

We recruited 45 patients. A total of 37 patients completed the study, reaching saturation (i.e., 13 PwS; 11 with PD; 11 with MS). Only two participants were undergoing post-operative neurosurgical rehabilitation for oncological conditions (Post-NS) (Table 2). Given the richness of the data provided by these participants and the relative rarity of this clinical population, their interviews were included in the analysis as illustrative cases contributing additional insights into the experience of neurorehabilitation hospitalization. The mean age of the sample was 61.1 years, and the average hospital stay was 82 days (Table 3). We excluded eight patients due to incomplete interviews or insufficient data.

The analysis led to the identification of 50 codes which were subsequently organized into five themes (Table 4). Through an iterative evaluation of the transcripts, regular discussions among the researchers, and consensus meetings, these themes were progressively refined to capture the core dimensions of participants’ experiences. The codes were used as analytical tools to support the organization and interpretation of meaningful statements, while the final thematic framework emerged from a deep collaborative reflection on participants’ lived experiences.

The names of the five themes were developed by the research team and reflect the conceptual understanding of the phenomena emerging from the interviews:

1. “The Therapeutic Bond” (experience with healthcare professionals);
2. “The Slow Time of Hospitalization” (hospitalization experience);
3. “Care is Rehabilitation” (care and rehabilitation as an integrated experience);
4. “Illness Narratives” (experience of illness and personal adaptation);
5. “Stories of Closeness Among Inpatients and Family Members” (experience with other patients and family members).

Theme 1: The Therapeutic Bond.

This theme focuses on experience with operators. Across interviews, professionalism, availability, support, and relational closeness emerged as key dimensions.

Physiotherapists are mentioned by all inpatients undergoing motor rehabilitation.

*“Sure, you can do physio all day long—maybe physically you’ll be fine—but if there’s something going on mentally… As physios say, you need to be in good headspace to do the physical stuff. They help you let things out and get through the day more calmly. And I mean everyone—the doctors, physios, psychologists, some of the care workers, the nurses. All of them make life here a bit easier.”*

(PwS 15)

One participant described the physiotherapist as both a coach and a partner.

*“To me, a physio is like a coach, but also a partner—and you don’t click with everyone. One time I had this girl [another physiotherapist], and people said, ‘Oh, she’s great.’ It wasn’t a total waste of an hour, but almost. She just wasn’t very tuned in, didn’t really listen.”*

(Post-NS 01)

Some interviewees described the relationship in negative terms, particularly highlighting a lack of availability. These comments were often referring to healthcare assistants (HCAs), the staff responsible for providing daily assistance.

*“Some people have a lot of it, some others too little… I mean humanity and heart. I get that we’re not the easiest patients—we’ve got limitations. Like me, for example: besides the limitations, I’m heavy, I’m big… Moving me, helping me with posture and stuff, it’s not easy. And sure, everyone wants to protect their back, take care of their own health”.*

(PwS 13)

The interactions with healthcare assistants appear to be of particular importance, especially for patients who require assistance with activities of daily living (ADLs) such as hygiene, mealtime support, or mobility in bed or a wheelchair.

*“We see the care workers way more, especially in the mornings when they help us wash. In my case, I need help to get going—changing my diaper at lunch, getting into bed. They come by at least four times a night. It’s a small kind of support, but it adds up. Rehab has to be full-on—mind and body, all of it”.*

(Post-NS 01)

Unlike more formally structured professional roles such as physiotherapists or nurses, the assistance provided by HCAs is described as varying in both quality and availability. Nonetheless, patients consider them highly important as they are the ones who meet their most intimate needs, primarily bodily, within what is often described as an *“emotional”* (PD02) relationship.

*“When it comes to care workers, I don’t know… it depends. Some are great, some not so much, it really comes down to the person’s sensitivity.”*

(PwS 15)

In patients’ experience, support is often perceived as an important component of professionalism, particularly in relation to assistance with ADLs, while also encompassing emotional and motivational dimensions.

“*Every day feels like starting from scratch, but with those couple of tips they give you [the operators], you really do get through the day better*.”

(PwS 15)

“*The physio is like a psychologist. A coach. Everything was going fine until last Friday. Then this thing with my back flared up… and the tears just came, the pain was intense. I could only handle two or three positions. But he saw how much I was struggling, all week long, he noticed*.”

(Post-NS 01)

Other healthcare professionals, such as nurses and physicians, often appear in the background of patients’ accounts. Nevertheless, despite not being central figures in their narratives, patients assign them a role of supervision and reassurance.

*“If I talk to a doctor, they can give you greater and more detailed reassurance.”*

(PwS 18)

*“…there are the nurses, and you can always ask them whatever you want—they will answer you.”*

(PD 07)

Theme 2: “The Slow Time of Hospitalization”.

This theme focuses on hospitalization experience. Hospitalization was often experienced as a slow and suspended time, shaped by institutional routines, waiting, and dependence on others. Rehabilitation activities and their scheduled appointments set the patients’ day:

*“At 8:15, I’m already down in the gym. I do all the movements like my therapist showed me—all the exercises, walking with the walker. Then I do half an hour on the bike to loosen up my legs a bit. And after that, it all starts over again. I sit here, sometimes push myself up and down the hallway. And then I wait for the next morning. It’s a long wait, always.”*

(PD 01)

Patients with reduced autonomy must first be washed and mobilized before they can attend therapy, but organizational schedules do not always align with the patients’ needs.

*“Some people are lucky and get therapy first thing in the morning. So at 7:30 they come in, get you out of bed, and off you go. Otherwise, you’re in bed till 9”.*

(PD 01)

Mealtimes, especially in the evening, which are scheduled according to the kitchen staff’s shifts, are a source of complaints from many of our interviewees.

*“When my friends call and ask, ‘You’ve already had dinner?’ I’m like, ‘Yeah, babe, here we eat at the crack of dawn… I don’t even know why.’ So yeah, dinner, then dinner again—the whole day kind of revolves around meals.”*

(PD 02)

As part of the institutional scheduling logistics, many patients are put to bed early to avoid overburdening the night shift staff.

*“Downsides? First of all, the schedule. For some reason, dinner’s at like 17:15… and last night they put me to bed at 19:30. So yeah, lights out at a set time. Then I’d maybe watch the news on my phone for a bit… and I’d just crash. But then, boom—wide awake at 1 a.m., like a cricket.”*

(Post-NS 01)

Theme 3: “Care is Rehabilitation”.

This theme focuses on care and rehabilitation experience. For most interviewees, care and rehabilitation constituted a single, integrated experience. Rehabilitation is understood as a broader process supported by professional competence, relational continuity, encouragement and acts of care.

Narratives relating to care were frequently articulated through references to rehabilitation activities and relationships with rehabilitation professionals, whereas references to HCAs primarily concerned support with personal hygiene, mobilization, and other everyday bodily needs.

*“[Interviewer: What did they say when they were washing you?] …just joking around… we laughed together, and that made the day a little nicer, yeah, definitely better.”*

(PwS 11)

In these accounts of care and rehabilitation experiences, the therapeutic relationship was described as a central and necessary element across the activities and interactions involved in daily rehabilitation.

*“When I came down, I was in pieces. I remember the girl—the physio—she said, ‘Okay, let’s start from scratch,’ like it was nothing, maybe… Maybe she did it to give me courage. I don’t really know why she did it.”*

(PD 12)

*“No, no, they’re amazing girls* [the HCAs], *I can’t say a bad word. They’ve got strong personalities, they’re there for me, they try to give you strength, to lift you up. ‘Come on, don’t give up!’ I really can’t complain—they’ve treated me well, all of them.”*

(MS 02)

Exploring more specifically the patients’ perspective on their rehabilitation journey, the personalization of their hospitalization emerges as a very important aspect, described by many patients as proof of attention to them as unique individuals.

*“She was able—while I was doing an exercise that, by the way, was going really well—to come up with… I could almost hear her brain going ‘mumble mumble mumble,’ like in cartoons, because she was thinking of a different exercise than the one I was doing. One that, in her opinion, would challenge me a bit. She needed to push me a little, because some people—like me—need to be challenged just enough. If it’s too much, it becomes pressure.”*

(PD 02)

The consistent presence of the same professional overseeing the rehabilitation process is considered a key factor in promoting both continuity and the overall effectiveness of rehabilitation.

*“Having the same physiotherapist all the time really helps, because they can see how you’re improving. Whereas someone who follows you consistently can really see your progress—whether you’re getting better or worse. Either way.”*

(PwS 18)

Theme 4: “Illness Narratives”.

This theme focuses on illness experience. The experience of illness emerged throughout the interviews, sometimes unconsciously and other times deliberately expressed by the patients during their narratives, although exploring the lived experience of illness was not the primary focus of the study. Participants frequently described illness as a disruptive life event that marked a transition between the former and the current self.

*“It’s clear—it’s a constant, slow decline. But I want to… since my life is really active, because I work, because I keep going, and I always want to do my best… before I give up on the things I have to give up, I need to be the one to realize it myself.”*

(MS 01)

In addition to acceptance, patients also expressed feelings of anger, frustration, and distress related to the loss of autonomy and previously taken-for-granted abilities:

“*Honestly, every time I need something, having to ask for it, not being able to do it myself… Oh my God, if I could tear down the wall, I’d tear the whole thing down*.”

(PwS 13)

“*But I forgot… can you believe I struggled to remember the names of my four grandkids, who I absolutely adore… They make me smile. And I couldn’t remember a thing. That really shook me up, honestly*.”

(PwS 10)

Among some individuals with PD, the illness experience is often marked by unpredictability and distress, especially during freezing episodes:

*“Yes, I’m scared of the symptom, because in my case it’s really intense… Try asking one of the HCAs or nurses who’ve seen me. I’m scary—I look completely out of it, like I don’t even have a brain. I can’t look people in the eyes anymore, I can barely speak. I can still see the world, but I just can’t connect with it—like I’ve got two fingers hanging onto the edge, and I can’t lift my arm back up.”*

(PD 10)

Theme 5: “Stories of Closeness Among Inpatients and Family Members”.

This theme focuses on experiences with other inpatients and family members. Relationships with other inpatients were often described in terms of mutual solidarity, both in practical matters and in emotional support.

*“So we’re happy, of course, because we help each other out. I’m the only one who can walk, so I kind of act as a go-between for the others in wheelchairs… So at night, if they need something, I’m the one who gets up. We look out for each other; it’s not something you find every day. It’s a real bond.”*

(PD 08)

*“Or we just chat about this and that, or about our lives outside the hospital. When we get comfortable enough to open up, we share stories, we tell each other what we used to do for work.”*

(PwS 18)

*“We go have coffee. Yeah, and reaching the end of the hallway is tiring, so it’d be better if it was closer. With the excuse of having coffee, you start talking about that person or this one. It creates a different kind of relationship.”*

(PwS 18)

Some patients, especially the more autonomous ones, complain about living with patients who have different characteristics and needs than them:

*“Here’s a thought: if it were possible to bring together people who have the same needs and problems, so they can see and understand how others handle different issues—what solutions others have found—that could be helpful and positive for me too.”*

(MS 03)

Visits from family members, relatives, and, at times, friends emerged as recurring themes in patients’ narratives and shaped the everyday experience of hospitalization. While some patients received frequent visits, for others they were less frequent yet remained meaningful and significant occasions.

*“But, like I said, I don’t demand anything from them or anyone. That is, the visit must be a pleasure, something to be felt in my opinion, so when you see someone you don’t expect it’s Easter* [it is to say that it’s a day of great celebration].

(PwS 18)

*“Whatever, but I feel different when my wife is here. When my wife is not here, I feel bad. I am used to a family. Without the family I feel lost.”*

(PwS 09)

## 4. Discussion

Qualitative analysis of interviews with patients with neurological disorders identified five key elements that constitute the main findings of this study. But beyond the specific themes identified, the findings highlight broader experiential dimensions of neurorehabilitation hospitalization. Across diagnostic groups, participants described their experiences of hospitalization and neurorehabilitation through relationships with healthcare professionals and fellow patients, changes in bodily autonomy, the temporal rhythms of institutional life, and the social environment of the ward. These dimensions suggest that neurorehabilitation hospitalization is experienced not only as a clinical intervention, but also as a relational, embodied, temporal, and social experience.

The first theme, the centrality of the therapeutic relationship, extending beyond clinical care to encompass emotional support and social interaction, emerged consistently across nearly all patient narratives, irrespective of diagnosis. Secondly, in general, patients did not rigidly distinguish between “medical care” and “rehabilitation”, instead perceiving them as integrated dimensions of care, where rehabilitation itself is part of medical care. A third key finding highlighted how variability in the quality of relationships with HCAs deeply influenced the patient experience, at times critically, particularly for those with reduced autonomy. Within this subgroup, a fourth theme emerged: for participants with reduced autonomy, the hospitalization experience seems to be largely shaped by two main dimensions: basic care and support for ADLs provided by HCAs, and the structured rehabilitative component.

1. “The Therapeutic Bond” (Theme 1)

Once patients began discussing the care provided by HCAs, they tended to share vivid, detailed stories and reflections. These narratives focused on basic but essential care, particularly hygiene, mobilization, and the management of minor daily needs such as drinking or elimination, which, in the ward setting, were primarily handled by HCAs. The participants’ accounts reveal a polarization in the perception of relational aspects: physiotherapists are described as highly technical-skilled professionals who nonetheless succeed in establishing personalized and meaningful relationships with patients. In contrast, HCAs are not perceived as “technical” figures, but rather as those who enter the patient’s intimate space, managing daily physical intimacy and the vulnerabilities associated with it. This closeness, however, also presents challenges. While the quality of the relationship with physiotherapists and other healthcare professionals appears consistent, the quality of the relationship with HCAs shows significant variability. This variability is largely dependent on individual personalities and is easily influenced by the organizational context [20,21]. This highlights potential considerations regarding the relational education and training of HCAs, while suggesting that future research could explore organizational structures that support the stability of relationship quality, particularly in the context of fundamental care [22,23].

These findings confirm and expand the recent literature. The therapeutic relationship has been identified as a significant predictor of rehabilitation outcomes, particularly in post-stroke care [20,24,25]. Our qualitative data show that even in other patient groups, the relationship is perceived as the cornerstone of professional care [24]. Indeed, professionalism is closely interconnected with relational competence. The patients’ narratives confirm what Jensen et al. identified as the hallmark of physiotherapy practice, the seamless integration of technical mastery with deep interpersonal connection [26]. As one stroke patient emphasized, “You can see they’re passionate, they know what they’re doing. I believe in the exercises they make me do” (PwS 10). The patient’s emphasis on both technical knowledge (“they know what they’re doing”) and affective engagement (“they’re passionate”) sustains findings from rehabilitation psychology on how therapeutic alliance enhances neurorehabilitation outcomes [27], while also aligning with nursing research on embodied care, an approach that integrates emotional, physical and ethical responsiveness in therapeutic encounters [28].

Patients’ descriptions of therapists as both “trainer and partner” (Post-NS 01) highlight the dual role that clinicians play in combining structured physical guidance with empathetic connection, a dynamic emphasized in nursing models of therapeutic presence and in psychology’s concept of working alliance [28,29]. The emphasis on continuity, “the only one who sees all your progress” (Post-NS 01), reflects the importance of sustained clinician–patient relationships [30,31].

Relational competences such as empathy, attunement, and continuity have been shown to directly influence clinical outcomes, as demonstrated in meta-analyses on therapeutic alliance in chronic illness and rehabilitation [32,33]. Their relevance cuts across disciplinary boundaries: systematic reviews in nursing, psychotherapy, and physiotherapy consistently identify alliance as a core predictor of effectiveness [34,35,36]. These findings suggest that the experience of rehabilitation is lived not only through therapeutic interventions, but also through the quality of the interpersonal relationships developed during hospitalization. Being listened to, supported, and understood emerges as a central component of the experience and, for some interviewees, of rehabilitation itself.

2. “The Slow Time of Hospitalization” and “Care is Rehabilitation” (Themes 2 and 3)

The two themes concerning the temporal dimension of hospitalization and the rehabilitation experience as a form of fundamental care have been merged, as both capture how patients experienced hospitalization and rehabilitation care as services delivered by the hospital. The experience of time emerged as a relevant aspect of hospitalization. Participants frequently described their days through narratives centered on routines, waiting periods, scheduled rehabilitation sessions, mealtimes, and anticipation of family visits. Hospitalization was therefore experienced through a temporal structure that differed substantially from the rhythms and routines of everyday life. Feelings of boredom emerged prominently across many narratives and were often associated with inactivity between rehabilitation sessions. Among participants with greater functional dependency, institutional routines frequently shaped the organization of daily activities and contributed to the perception of prolonged, repetitive, and, at times, monotonous time.

Another interesting finding is the limited mention of physicians and nurses in patients’ narratives, with these professionals often being perceived, when discussed, as more focused on clinical aspects. This does not imply their irrelevance; indeed, some interviews highlighted their role in supervising the care pathway, but it is another indicator of how the perception of “care” is primarily shaped by daily interactions with the most present and accessible staff [37]. Conversely, the central role of physiotherapists and HCAs in patients’ experience emerged clearly: besides the distinction between rehabilitation activities and fundamental care, patients did not experience them as separate activities. This means that care processes are perceived as a fundamental part of the rehabilitation experience.

3. Illness Narratives (Theme 4)

In qualitative studies, PwSs described dependence on others for personal hygiene or mobility as emotionally distressing and isolating [38,39]. Kitson et al. described the experience of PwSs receiving basic care (hygiene, elimination, nutrition, hydration, etc.) as frustrating and humiliating, often associated with a loss of dignity and self-esteem [21]. The patients in question are those with low levels of autonomy: they are highly dependent on healthcare staff, perceive time as slow and drawn out, and passively endure the boredom and repetitiveness of the hospital routine which follows rigid institutional scheduling practices structured around HCAs, physiotherapy sessions, meals, being helped out of and back into bed, and medical treatments while waiting for family visits [40]. This further underscores how patients often attribute value to competent and empathetic HCAs, particularly in relation to dignity and autonomy. These findings indicate potential implications for how nursing activities in a neurorehabilitation setting may evolve, particularly regarding comprehensive patient assessment and the supportive supervision of healthcare assistants (HCAs), representing key areas for future organizational research.

The embodied nature of the neurorehabilitation experience also emerged from the findings. Bodily needs, such as dependence on others for mobility, personal hygiene, dressing, and other ADLs, became central aspects of everyday life, often associated with feelings of vulnerability, a loss of autonomy, and concerns about personal dignity [40].

4. Stories of Closeness Among Inpatients and Family Members (Theme 5)

An area that deserves further in-depth investigation is the social and peer relationship developed among inpatients, which appears to play a supportive role in the hospitalization experience. Sharing one’s condition fosters mutual understanding and emotional support, while engaging in everyday conversations, especially in less clinical settings such as gardens or communal break areas; it helps counteract the boredom and isolation that often characterize prolonged inpatient stays [5]. These relational dynamics, however, are not without complexity: some patients report difficulties in sharing space with individuals who have significantly different levels of autonomy or who manifest intense suffering that may provoke physical or emotional discomfort. These interactions, while frequently a source of comfort, can also become a site of emotional vulnerability. This dual nature aligns with the existing literature highlighting the central role of the social environment in rehabilitation contexts, and how such environments may significantly affect both health and rehabilitation outcomes [4,41,42].

These findings suggest that the rehabilitation ward may function not only as a site of treatment but also as a social environment in which relationships and shared experiences contribute substantially to how hospitalization is perceived and experienced by patients.

To our knowledge, no qualitative studies have explored hospitalization experiences across different neurorehabilitation populations so far. The present study contributes to the literature by examining common experiential dimensions among PwS, MS, PD and, although only exploratorily, post-neurosurgical patients within the same rehabilitation setting.

This is also one of the few studies that highlights the crucial yet underestimated role of HCAs, as perceived by patients. HCAs were perceived by participants as playing an essential role in addressing physical needs, particularly those related to basic care, as well as providing emotional support; however, the quality of their interactions is perceived to vary considerably.

Taken together, these findings suggest that neurorehabilitation hospitalization extends beyond the mere delivery of clinical interventions. Participants described an experience shaped by relationships, bodily vulnerability, institutional routines, and the social environment of the ward, highlighting the potential value of person-centered models of care as a relevant framework for further investigation.

### Limitations

This study has several methodological limitations. Besides the sample being heterogeneous in terms of conditions, it was limited to a single neurorehabilitation center. Participation was voluntary and therefore self-selection bias cannot be excluded. Additionally, the researcher’s role as interviewer may have influenced participant interactions, despite the implementation of measures to ensure neutrality in data collection and analysis [43]. Moreover, patients with cognitive impairments and/or severe aphasia were not included in the study. This may have limited the range of experiences represented, as well as the visibility of specialized professional roles. Only two participants undergoing post-operative neurosurgical rehabilitation following oncological conditions were included. Recruitment of this population was particularly challenging, as these patients often followed complex clinical trajectories characterized by emotional distress, short rehabilitation admissions, and frequent transitions to further neurosurgical procedures or adjuvant oncological treatments. Consequently, some did not meet the study inclusion criteria, whereas others declined participation, limiting the number of eligible participants available for recruitment. Although these interviews were retained because of the richness of the narratives and the relative rarity of this clinical population, findings related to this subgroup should be considered exploratory and should not be generalized to the broader post-neurosurgical population.

Even if some of the experiential dimensions identified may be transferable to similar rehabilitation settings, however, local organizational aspects and specific healthcare system characteristics should be considered when interpreting the findings.

## 5. Conclusions

The aim of this study was to explore and describe the care and hospitalization experience of individuals admitted to neurorehabilitation units. Across all diagnostic groups, rehabilitation has a central role. In our study, patients did not distinguish clearly between “medical care” and “rehabilitation”. Rather, they described these as a single, relationally rich process in which even small daily interactions and expressions of support held significant meaning.

These findings reinforce the importance of considering patients’ experiences as an integral component of neurorehabilitation hospitalization. In particular, the therapeutic relationship, daily interactions with healthcare professionals, including healthcare assistants, and the broader organizational and social context of hospitalization emerged as relevant aspects of patients’ experiences [41]. Future research should further explore how these dimensions may contribute to the development of more person-centered approaches to neurorehabilitation.

## Figures and Tables

**Table 1:** Interview questions.

N°	Interview Questions
1	Can you tell me the reason for your admission to this hospital?
2	Can you describe a typical day for you?
3	How would you describe your hospitalization experience?
4	What has been your experience with the care provided?
5	What has been your experience with rehabilitation?
6	Does the experience meet your expectations?
7	Can you describe any particularly meaningful events from this experience?
8	Do you have anything to add?

**Table 2:** Demographic and clinical characteristics of sample.

ID	Sex	Age	Family Network	Diagnosis	Previous Neurorehab Admissions	Barthel Index
STR_01	F	50–59	yes	Right ischemic stroke, left hemiparesis.	yes	Admission: 65 Discharge: 95
STR_02	F	50–59	yes	Right ischemic stroke, left hemiparesis	no	Admission: 60 Discharge: 80
STR_03	F	30–39	yes	Right hemorrhagic stroke, Left hemiparesis	yes	Admission: 20 Discharge: 40
STR_06	M	50–59	yes	Left ischemic stroke, right hemiparesis	no	Admission: 20 Discharge: 40
STR_09	M	70–79	yes	Right ischemic stroke, left hemiparesis,	yes	Admission: 30 Discharge: 55
STR_10	M	70–79	yes	Left ischemic stroke, left hemiparesis	no	Admission: 0 Discharge: 45
STR_11	F	50–59	yes	Left ischemic stroke, right hemiparesis	no	Admission: 70 Discharge: 90
STR_12	F	60–69	yes	Left hemorrhagic stroke, right hemiparesis	yes	Admission: 54 Discharge: 70
STR_13	M	40–49	yes	Right hemorrhagic stroke, left hemiparesis	yes	Admission: 50 Discharge: 30
STR_15	M	20–29	yes	Right hemorrhagic stroke subsequent to traumatic brain injury, left hemiparesis	yes	Admission: 65 Discharge: 70
STR_16	F	80–89	yes	Subarachnoid left hemorrhage, right hemiparesis	yes	Admission: 20 Discharge: 40
STR_17	M	80–89	yes	Left ischemic stroke, right hemiparesis	yes	Admission: 25 Discharge: 50
STR_18	M	60–69	yes	Right hemorrhagic stroke, left hemiparesis	yes	Admission: 5 Discharge: 35
P/01	M	70–79	yes	Parkinson’s disease with gait impairment	yes	Admission: 25 Discharge: 60
P/02	F	50–59	no	Advanced Parkinson’s disease with motor fluctuations, dyskinesias, and motor blocks	yes	Admission: 60 Discharge: 95
P/03	F	50–59	yes	Parkinson’s disease with bradykinesia, gait difficulties, freezing of gait, and generalized motor impairment	no	Admission: 50 Discharge: 90
P/05	M	70–79	yes	Parkinson’s disease with significant reduction in motor abilities and autonomy in ADLs	yes	Admission: 50 Discharge: 65
PA_06	F	80–89	yes	Parkinson’s disease (with ADL impairment)	no	Admission: 60 Discharge: 95
PA_07	M	70–79	yes	Parkinson’s disease (with mild ADL impairment)	no	Admission: 60 Discharge: 100
PA_08	M	70–79	yes	Parkinson’s disease with initial wearing-off phenomena	no	Admission: 80 Discharge: 90
PA_09	M	50–59	yes	Parkinson’s disease (post-operative complications after recent DBS placement)	no	Admission: 60 Discharge: 100
PA_10	M	50–59	yes	Parkinson’s disease with disabling motor fluctuations	yes	Admission: 60 Discharge: 95
PA_12	M	50–59	yes	Parkinson’s disease (DBS implant)	yes	Admission: 65 Discharge: 90
PA_13	F	70–79	yes	Parkinson’s disease (DBS implant)	yes	Admission: 50 Discharge: 85
SM_01	M	50–59	yes	Multiple sclerosis	yes	Admission: 30 Discharge: 55
SM_02	F	60–69	yes	Multiple sclerosis	yes	Admission: 30 Discharge: 45
SM_03	F	50–59	yes	Multiple sclerosis	yes	Admission: 30 Discharge: 55
SM_04	F	50–59	yes	Multiple sclerosis	yes	Admission: 40 Discharge: 65
SM_05	F	60–69	yes	Multiple sclerosis	yes	Admission: 30 Discharge: 50
SM_06	M	60–69	yes	Multiple sclerosis	yes	Admission: 40 Discharge: 50
SM_07	F	70–79	yes	Multiple sclerosis	yes	Admission: 40 Discharge: 60
SM_08	F	70–79	yes	Multiple sclerosis	yes	Admission: 95 Discharge: 95
SM_09	F	50–59	yes	Multiple sclerosis	yes	Admission: 60 Discharge: 70
SM_10	F	60–69	yes	Multiple sclerosis	yes	Admission: 50 Discharge: 70
SM_11	F	30–39	yes	Multiple sclerosis	yes	Admission: 80 Discharge: 90
Post-NS_01	F	50–69	yes	Post-neurosurgical rehabilitation following an oncological condition	no	Admission: 25 Discharge: 45
Post-NS_03	F	40–69	yes	Post-neurosurgical rehabilitation following an oncological condition	no	Admission: 80 Discharge: 90

**Table 3:** Descriptive statistics of sample.

Demographic and Clinical Characteristics		
Sex	Mean	
F	18 (48.6%)	
M	19 (51.4%)	
Age	Mean (SD)	Range
	61.1 (14.0)	28.0–85.0
Family support	Yes	No
Yes	36 (97.3%)	1 (2.7%)
No	1 (8.1%)	
Comorbidities	Mean (SD)	Range
	0.6 (0.9)	0.0–4.0
Barthel Index (Admission)	Mean (SD)	Range
	48.3 (21.7)	0.0–95.0
Barthel Index (Discharge)	Mean (SD)	Range
	67.3 (23.1)	25.0–100.0
Barthel Index difference (Discharge–Admission)	Mean (SD)	Range
	19.0 (15.3)	−30.0–40.0
Average hospital stay (days)	Mean	Range
	82	50–157

**Table 4:** Themes and essential meanings emerging from participants’ experiences.

Theme	Essential Meaning	Illustrative Quotation
The Therapeutic Bond	Patients described professionalism through empathy, availability, continuity of care, and supportive relationships with healthcare professionals.	“The physio is like a psychologist. A coach… But he saw how much I was struggling, all week long, he noticed.” (Post-NS 01)
The Slow Time of Hospitalization	Hospitalization was experienced through routines, waiting, dependency, and the rhythm imposed by institutional schedules.	“At 8:15, I’m already down in the gym… And then I wait for the next morning. It’s a long wait, always.” (PD 01)
Care is Rehabilitation	Care and rehabilitation were often described as interconnected aspects of the same experience, supported by professional relationships and everyday acts of assistance.	“No, no, they’re amazing girls… They try to give you strength, to lift you up. ‘Come on, don’t give up!’” (MS 02)
Illness Narratives	Patients described illness through experiences of adaptation, loss, uncertainty, anger, acceptance, and hope.	“Honestly, every time I need something, having to ask for it, not being able to do it myself…” (PwS 13)
Stories of Closeness Among Inpatients and Family Members	Relationships with fellow patients and family members contributed to support, companionship, and meaning during hospitalization.	“We look out for each other… it’s not something you find every day. It’s a real bond.” (PD 08)

## Data Availability

The original contributions presented in this study are included in the article. Further inquiries can be directed to the corresponding author.

## Supplementary Materials

The following supporting information can be downloaded at: https://www.mdpi.com/article/10.3390/clinpract16100177/s1, File S1: COREQ checklist.
